# Supplementary material for: Detailed analysis and comparison of different activity metrics
Source: PLoS One. 2021 Dec 21;16(12):e0261718. doi: 10.1371/journal.pone.0261718 (PMC8691611; doi:10.1371/journal.pone.0261718)
Supplement: S1 Appendix — (PDF) [file pone.0261718.s002.pdf]

S1 Appendix – Comparison of the integration methods

Since the integration method used for calculating the PIM metric is not clearly stated in the literature, we examined two possible methods. The first and simpler one is the basic summation of data points (Riemann sum), and the other, more precise method is Simpson's 3/8 rule. We calculated every possible activity signal made by the PIM metric with both integration methods.

Simple Riemann Sum

**Simpson's 3/8 Rule**	PIM(|FMpost|)	PIM(UFM)	PIM(UFNM)	PIM(FMpre)	PIM(|FX|)	PIM(|FY|)	PIM(|FZ|)
PIM(|FMpost|)	1	0.79	0.99	0.92	0.9	0.94	0.84
PIM(UFM)	0.79	1	0.85	0.73	0.72	0.75	0.66
PIM(UFNM)	0.99	0.85	1	0.92	0.9	0.93	0.85
PIM(FMpre)	0.92	0.73	0.92	1	0.98	0.97	0.97
PIM(|FX|)	0.9	0.72	0.9	0.98	1	0.94	0.94
PIM(|FY|)	0.94	0.75	0.93	0.97	0.94	1	0.91
PIM(|FZ|)	0.84	0.66	0.85	0.97	0.94	0.91	1

SFig. 1 Correlation between activity signals calculated from each of the datasets to which PIM metric can be applied while using two different integration methods. The Pearson's correlation coefficients are calculated by 42 measurements, and their means are represented.

The diagonal values of the correlation matrix are all equal to 1 (rounded to two decimal places), while the smallest value of these elements is 0.99998. It can be said that the PIM metric does not depend on the chosen integration method. Therefore, in our further analyses, we used the simple numerical integral since it is easier to calculate with.
