# Supplementary material for: Detailed analysis and comparison of different activity metrics
Source: PLoS One. 2021 Dec 21;16(12):e0261718. doi: 10.1371/journal.pone.0261718 (PMC8691611; doi:10.1371/journal.pone.0261718)
Supplement: S2 Appendix — (PDF) [file pone.0261718.s003.pdf]

# S2 Appendix – Applicability of the activity metrics

In the following section, we will discuss the conditions of the applicability of the activity metrics listed in the article. For each activity metric, we examined whether they can or cannot be applied to a given dataset. The reasons limiting the applicability and the correctness of the application are illustrated through timeseries. All the activity signals presented in the following figures were calculated from the same time slice of the same measurement, therefore the procedure allows us to visually compare the resulting activity signals.

## *Directly applicable activity metrics*

Firstly, we will take a look at those activity metrics that gave correct results when we directly applied them to the given dataset. For these methods, neither the dataset nor the activity signal required further correction to obtain a correct result. The metric and dataset pairs are shown on SFig. 1 and are summarized in the following list:

- PIM (Proportional Integration Method): UFNM, FMpre
- Zero Crossing Method (ZCM): FXYZ, UFNM, FMpost, FMpre
- Time Above Threshold (TAT): FXYZ, UFNM, FMpost, FMpre
- Mean Amplitude Deviation (MAD): UFXYZ, FXYZ, UFM, UFNM, FMpost, FMpre
- Euclidian Norm Minus One (ENMO): UFM
- High-pass Filtered Euclidian Norm (HFEN): Requires its own specially pre-processed dataset.

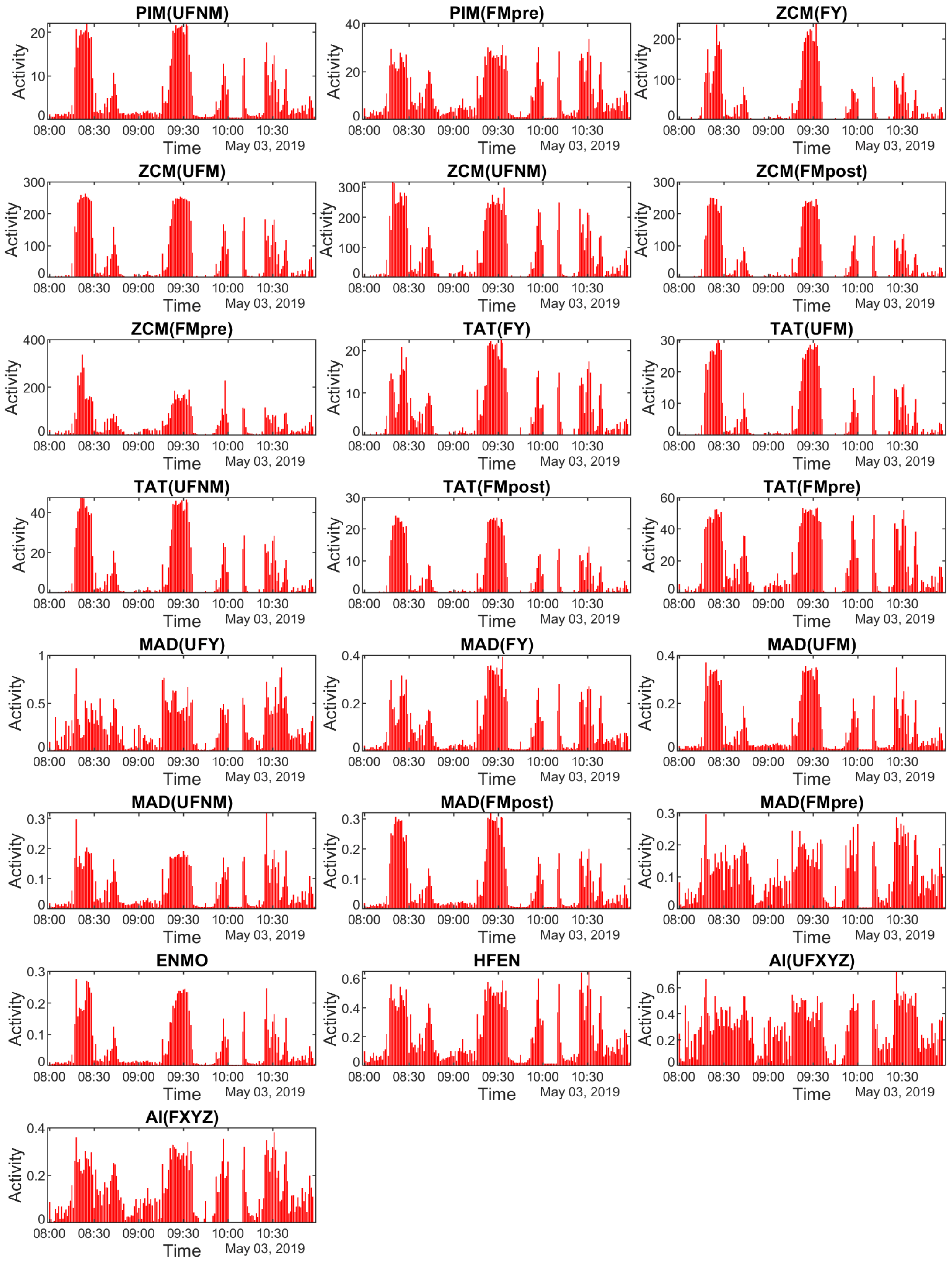


**SFig 1. Examples for the activity signals generated by every directly applicable activity metric.** In the case of axial datasets, only the y-axis is represented (UFY, FY) to reduce the figure size.

## *Indirectly applicable or inapplicable activity metrics*

In the following, we will present the cases when the activity metrics could not be applied directly to a given dataset. These activity metric and dataset pairs can be divided into two subgroups. Those that can produce interpretable activity signals by correcting the input dataset or the generated activity signal in some way, and the rest of them, whose application was limited by an insurmountable factor.

PIM (Proportional Integration Method)

- **UFXYZ dataset**:
  - **Statement**: The PIM metric is inapplicable on the UFXYZ dataset.
  - **Reason**: If no acceleration other than the gravity of Earth acts on the actigraph, the length of the resulting vector should be 1 g. However, for one axis, the measured acceleration depends on the orientation of the device as the gravity of Earth is distributed among the three axes. The PIM metric integrates that single axial acceleration. As a result, it creates plateaus with varying magnitudes (with 60 s epoch length in the range of [-60, 60]). Since we do not have information about the orientation of the device, these plateaus cannot be corrected. An example for this phenomenon can be seen in the following figure in the case of the y-axis.

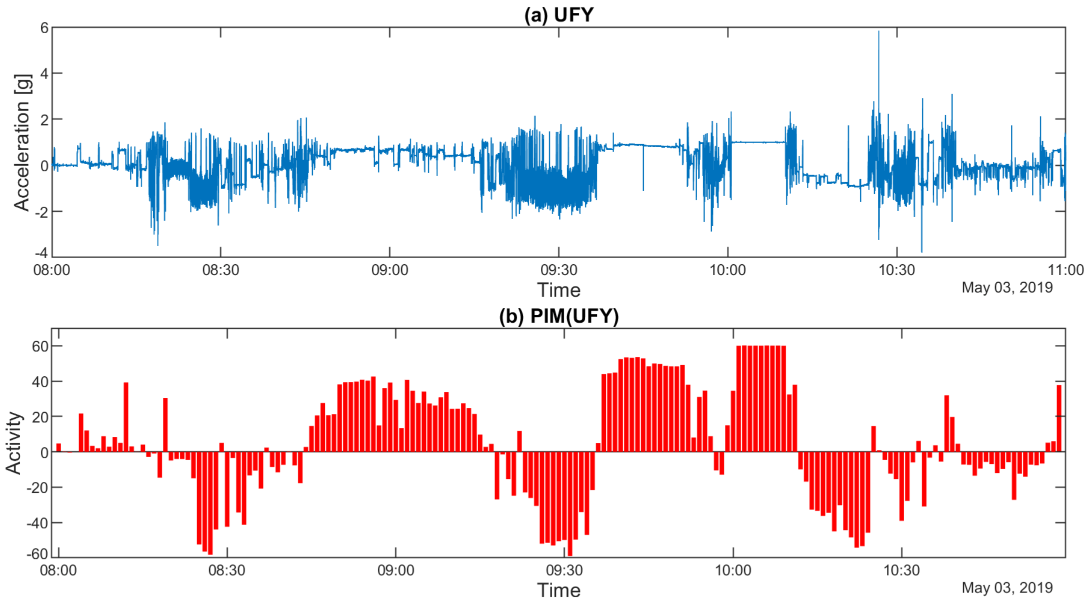


**SFig 2. An example for the application of PIM metric on the UFY dataset.** A section of a measurement's UFY dataset is presented in plot (a). The activity signal generated from this dataset by PIM metric is illustrated in plot (b).

- **FXYZ dataset**:
  - **Statement**: The PIM metric is applicable on the FXYZ dataset if we take the absolute values of the dataset before applying the metric.
  - **Reason**: Due to the bandpass filtering process, the values in the dataset are centralized around 0 g. The PIM metric integrates the filtered axial acceleration. However, sections with positive acceleration are always followed by the approximately same amount of negative accelerations. Therefore, the generated activity values are approximately equal to 0. This can be corrected if we take the absolute values of the values in the dataset, so it will contain only positive values. An example of this problem and solution can be seen in the following figure.

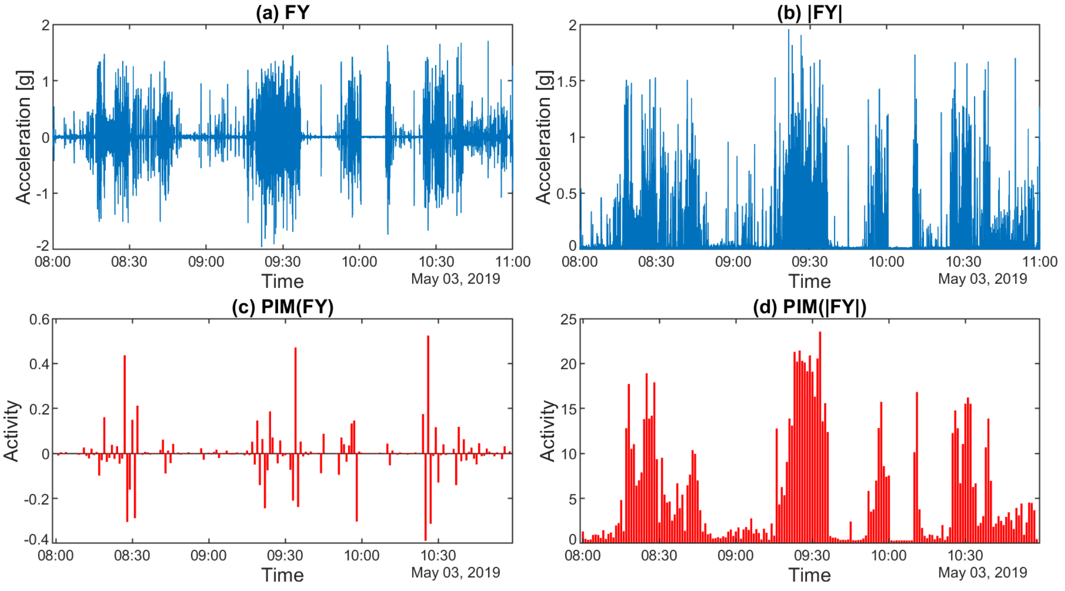


**SFig. 3 An example for the application and necessary corrections of PIM metric on the FY dataset.** A section of a measurement's UFY dataset is presented in plot (a) and its absolute values in plot (b). The activity signals generated from these datasets by PIM metric are illustrated in plots (c) and (d) in the same order.

- **FMpost dataset**:
  - **Statement**: The PIM metric is applicable on the FMpost dataset if we take the absolute values of the dataset before applying the metric.
  - **Reason**: The reason behind this correction is the same as the explanation we have already clarified in the case of the FXYZ dataset. An example of this problem and solution can be seen in the following figure.

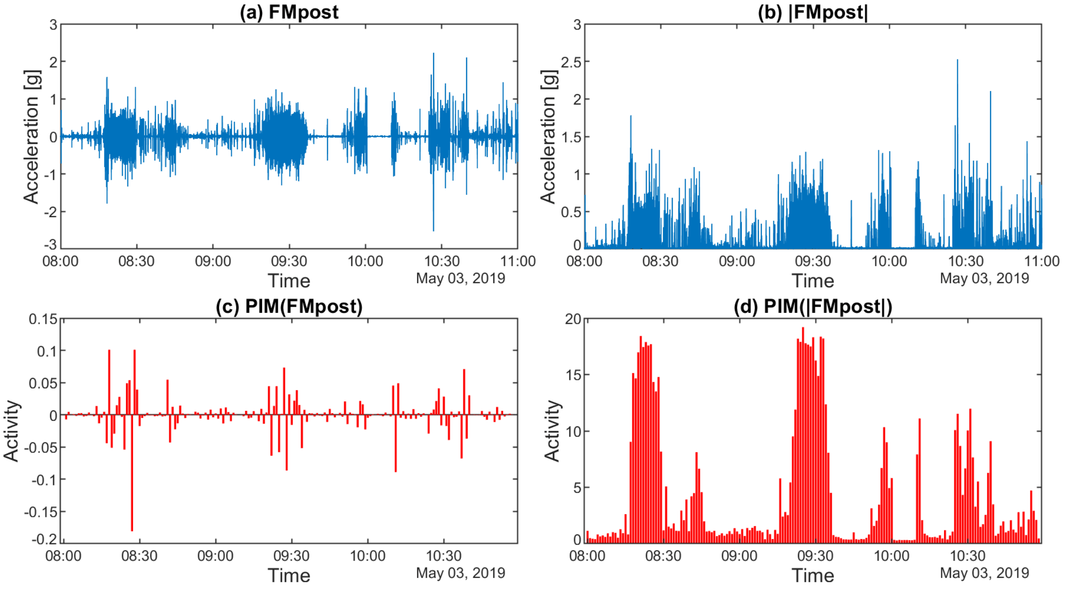


**SFig 4. An example for the application and necessary corrections of PIM metric on the FMpost dataset.** A section of a measurement's FMpost dataset is presented in plot (a) and its absolute values in plot (b). The activity signals generated from these datasets by PIM metric are illustrated in plots (c) and (d) in the same order.

- **UFM dataset**:
  - **Statement**: The PIM metric is applicable on the UFM dataset if we subtract the integral of the gravity of Earth from each activity value. The subtracted value is equal to the integral of 1 g over the epoch. To ensure that there will be no negative activity values, we have to take the absolute value of the difference.
  - **Reason**: Without bandpass filtering or normalizing, the length of the resultant acceleration contains the constant 1 g. When we apply the PIM metric on a UFM dataset, the gravity of Earth is integrated into the resulting activity value, too. However, this component is independent of the activity of the observed person, therefore it needs to be subtracted. An example of this problem and solution can be seen in the following figure.

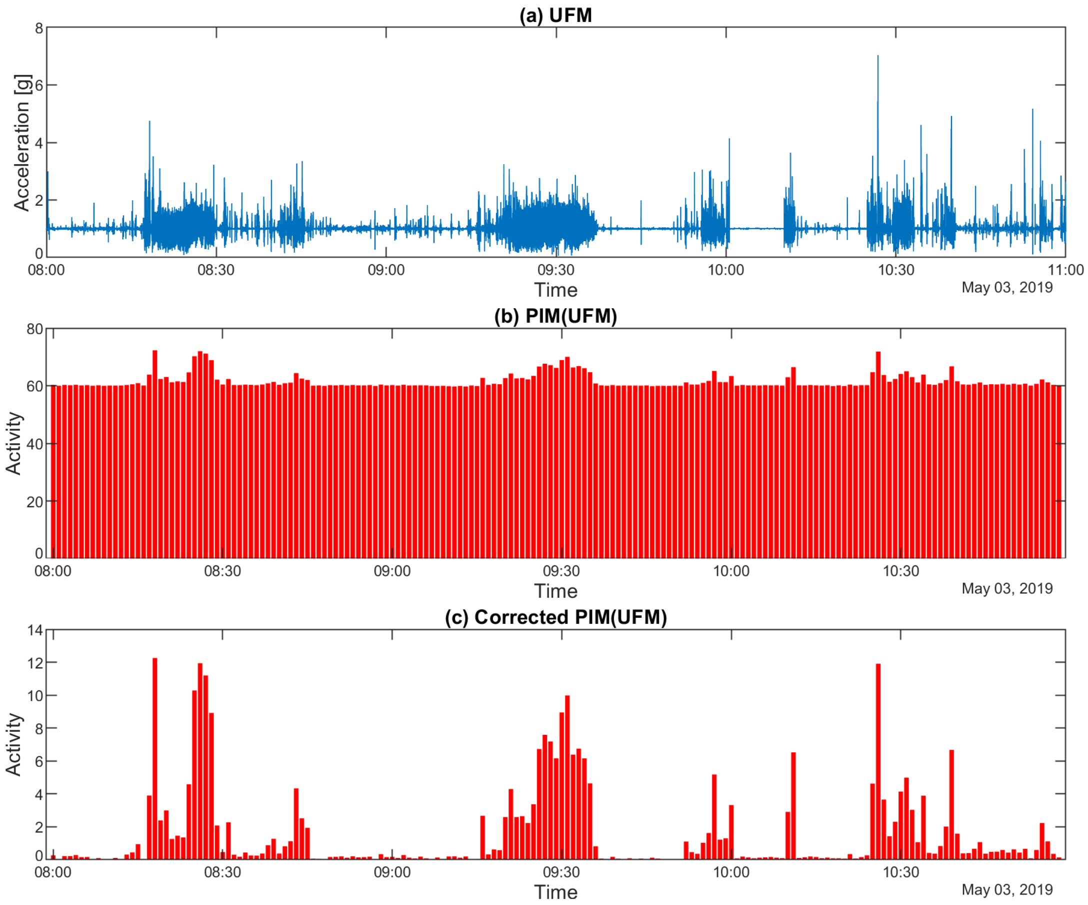


**SFig 5. An example for the application and necessary corrections of PIM metric on the UFM dataset.** A section of a measurement's UFM dataset is presented in plot (a). The activity signals generated from these datasets by PIM metric are illustrated in plots (b) and (c).

## Zero Crossing Method (ZCM) and Time Above Threshold (TAT)

The production of ZCM and TAT activity signals is based on a common thresholding principle. Therefore, the usability of these metrics is limited by the same issue, so we discuss these metrics together.

- **UFXYZ dataset**:
  - **Statement**: The ZCM and the TAT metric are inapplicable on the UFXYZ dataset.
  - **Reason**: As we mentioned earlier, at the use of PIM on the UFXYZ dataset: if no acceleration other than the gravity of Earth acts on the actigraph, the length of the resulting vector should be 1 g. However, for one axis, the measured acceleration depends on the orientation of the device as the gravity of Earth is distributed among the three axes. Therefore, the dataset may contain parts where the acceleration values remain unchanged. In the case of ZCM, it is theoretically possible that the selected threshold is equal to the magnitude of this constant part of the acceleration signal. Then the small fluctuations (noise) of the acceleration signal will generate activity values that are independent of the movement of the observed person. In the case of TAT, if the selected threshold is below the magnitude of the acceleration signal's constant part, it will generate false activity values even if no acceleration was recorded related to the movement of the observed person. Since we have no information about the orientation of the actigraph, we do not have the possibility to adaptively adjust the threshold values. An example of this problem can be seen in the following figure in the case of the y-axis.

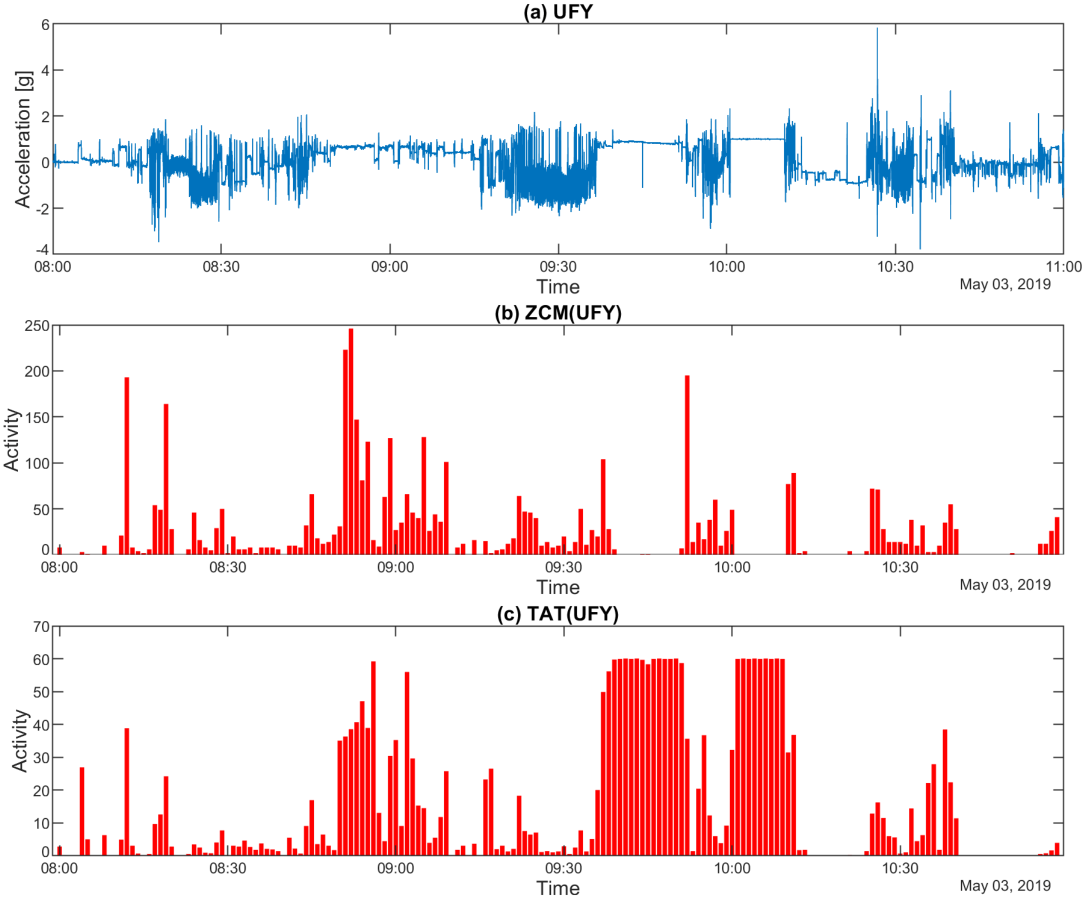


**SFig 6. An example for the application of ZCM and TAT metrics on the UFY dataset.** A section of a measurement's UFY dataset is presented in plot (a). The activity signals generated from these datasets by ZCM and TAT metrics are illustrated in plots (b) and (c) in the same order.

## Remaining metrics to mention

- **ENMO**: This metric eliminates the effect of gravity of Earth from the resulting acceleration values by definition. Therefore, this metric can be used only on those datasets that are built up by the magnitudes of the resultant acceleration vectors that contain the gravity of Earth. The only dataset that satisfies this requirement is the UFM dataset.
- **HFEN**: This metric requires a specially conditioned dataset, which is different from the six previously mentioned main types. The method of preprocessing can be found in the main article.
- **AI**: This metric requires the triaxial acceleration signals separately, therefore it cannot be used on datasets that are built by the magnitude of acceleration. The datasets which are satisfying this requirement are the UFXYZ and FXYZ datasets.
