# Supplementary material for: Detailed analysis and comparison of different activity metrics
Source: PLoS One. 2021 Dec 21;16(12):e0261718. doi: 10.1371/journal.pone.0261718 (PMC8691611; doi:10.1371/journal.pone.0261718)
Supplement: S3 Appendix — (PDF) [file pone.0261718.s004.pdf]

S3 Appendix – Further details of level crossing analysis

As we have mentioned in S2 Appendix, the ZCM and TAT metrics are directly applicable on the FXYZ and FMpost datasets. However, both of these datasets contain negative acceleration values. These values are centralized around 0 g due to the bandpass filtering process, which eliminates the DC component (gravity of Earth). Moreover, in the case of these datasets, the sign of the acceleration values depends on the orientation of the actigraph. Therefore, we examined if taking the absolute values (full-rectification) of the datasets makes any difference in the resulting activity values created by the ZCM or TAT activity metrics. During this process, we used the standard deviation of the given input data as the threshold value. The average Pearson's correlation coefficients of the activity signals are calculated by 42 different measurements and are presented in the matrices below.

(a)

	ZCM(FX)	ZCM(FY)	ZCM(FZ)	ZCM(|FX|)	ZCM(|FY|)	ZCM(|FZ|)	ZCM(FMpost)	ZCM(|FMpost|)
ZCM(FX)	1	0.93	0.95	0.97	0.94	0.95	0.96	0.93
ZCM(FY)	0.93	1	0.92	0.92	0.97	0.92	0.97	0.9
ZCM(FZ)	0.95	0.92	1	0.94	0.93	0.98	0.94	0.92
ZCM(|FX|)	0.97	0.92	0.94	1	0.95	0.96	0.94	0.95
ZCM(|FY|)	0.94	0.97	0.93	0.95	1	0.95	0.96	0.95
ZCM(|FZ|)	0.95	0.92	0.98	0.96	0.95	1	0.94	0.94
ZCM(FMpost)	0.96	0.97	0.94	0.94	0.96	0.94	1	0.95
ZCM(|FMpost|)	0.93	0.9	0.92	0.95	0.95	0.94	0.95	1

(b)

	TAT(FX)	TAT(FY)	TAT(FZ)	TAT(|FX|)	TAT(|FY|)	TAT(|FZ|)	TAT(FMpost)	TAT(|FMpost|)
TAT(FX)	1	0.94	0.94	1	0.94	0.94	0.91	0.92
TAT(FY)	0.94	1	0.92	0.94	1	0.92	0.95	0.95
TAT(FZ)	0.94	0.92	1	0.94	0.92	1	0.86	0.87
TAT(|FX|)	1	0.94	0.94	1	0.95	0.95	0.91	0.92
TAT(|FY|)	0.94	1	0.92	0.95	1	0.93	0.95	0.95
TAT(|FZ|)	0.94	0.92	1	0.95	0.93	1	0.87	0.88
TAT(FMpost)	0.91	0.95	0.86	0.91	0.95	0.87	1	1
TAT(|FMpost|)	0.92	0.95	0.87	0.92	0.95	0.88	1	1

SFig 1. Correlation between activity signals calculated from the FMpost, FY datasets, and their full-rectified pairs in the case of ZCM (a) and TAT (b) metrics. The Pearson's correlation coefficients are calculated by 42 measurements, and their means are represented. The relevant cells, which indicate the effect of full-rectification, are colored with blue (ZCM) and yellow (TAT).

The correlation matrices reveal that the full-rectification of the FY and FMpost datasets does not impact the generated activity signals of the ZCM and TAT metrics in the sense of linear relationships. In the case of the ZCM metric, the relevant correlation coefficients are all greater than or equal to 0.95 if rounded to two decimal places. In the case of the TAT metric, the relevant correlation coefficients are all equal to 1 if rounded to two decimal places (the smallest value of them is 0.99675).

We also inspected the shape of the resulting activity signals. An example can be seen in the following figure, where we compared the activity signal of the ZCM metric applied on an original FY and a full-rectified FY dataset.

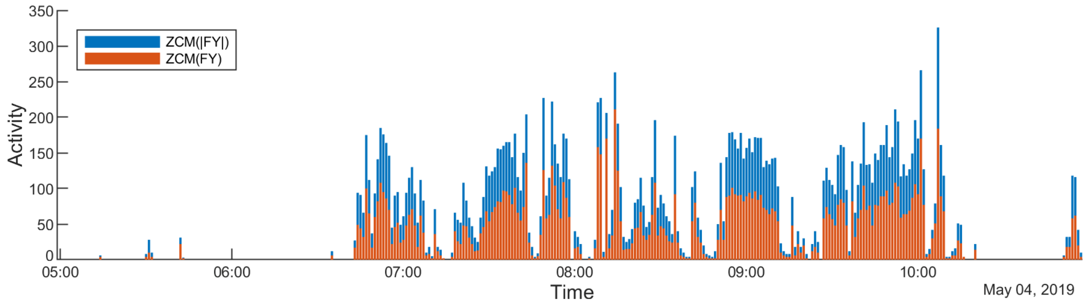

SFig 2. An example for the difference between the generated activity signals by the ZCM metric based on the FY dataset and on the full-rectified FY dataset.

The visible differences between the two time series confirm the findings based on the correlation matrices. The two activity signals track each other, but the full-rectification approximately doubles the generated activity values. The same is true for the other metric and dataset combinations marked in the SFig 2 correlation matrices. Therefore, it is unnecessary to take the absolute values of these datasets before applying the ZCM or TAT metrics.

Threshold value for the ZCM and TAT metrics

In the article, Fig 5 and Fig 6 present the results for a dataset containing vector magnitudes (UFM) and a dataset containing axial accelerations (FY) as examples, but in this following figure, the additional graphs for the remaining datasets are illustrated for both ZCM and TAT metrics.

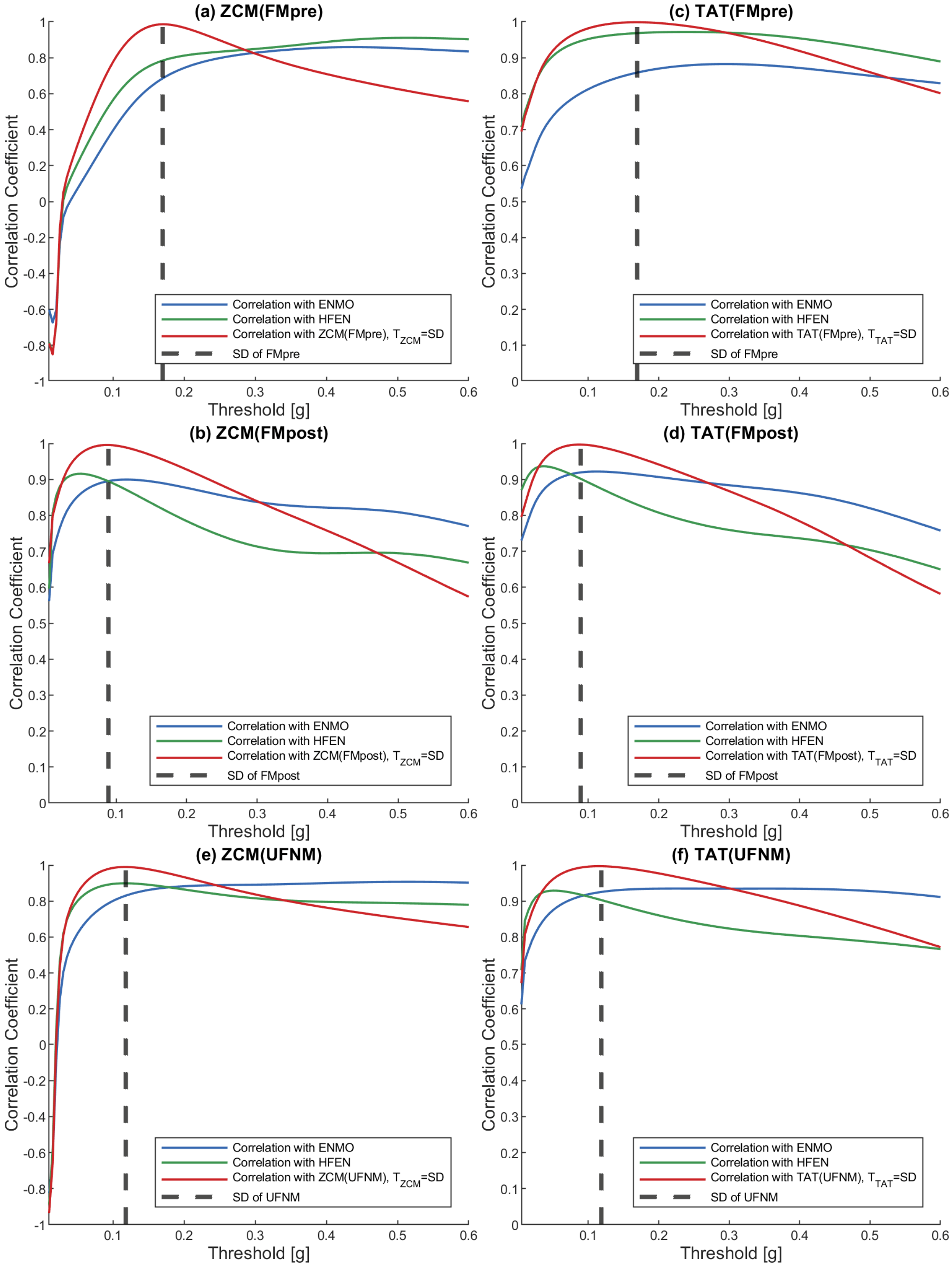

SFig 3. Extension of the article's Fig 5 and Fig 6.
