# Supplementary material for: Detailed analysis and comparison of different activity metrics
Source: PLoS One. 2021 Dec 21;16(12):e0261718. doi: 10.1371/journal.pone.0261718 (PMC8691611; doi:10.1371/journal.pone.0261718)
Supplement: S4 Appendix — (PDF) [file pone.0261718.s005.pdf]

S4 Appendix – Spectral correlation of activity signals

In the article, Fig 8 presents the time-domain correlation between activity signals calculated from raw datasets (UFM, UFXYZ) and filtered datasets (FMpre, FXYZ). In addition to this, the following figure presents the same analysis, but in frequency-domain, where we compared the power spectral densities of the activity signals. The means and standard deviations of the correlation coefficients of the different activity signals' power spectral densities are also presented in the S1 Table.

	PIM(UFM)	PIM(UFNM)	ZCM(UFM)	TAT(UFM)	MAD(UFM)	AI(UFXYZ)	ENMO	HFEN	PIM(FMpre)	ZCM(FMpre)	TAT(FMpre)	MAD(FMpre)	AI(FXYZ)
PIM(UFM)	1	0.92	0.85	0.89	0.92	0.75	0.96	0.88	0.87	0.79	0.84	0.81	0.85
PIM(UFNM)	0.92	1	0.95	0.98	1	0.81	0.99	0.96	0.95	0.86	0.94	0.87	0.92
ZCM(UFM)	0.85	0.95	1	0.98	0.95	0.81	0.93	0.95	0.95	0.91	0.96	0.86	0.91
TAT(UFM)	0.89	0.98	0.98	1	0.97	0.8	0.96	0.95	0.95	0.88	0.95	0.86	0.91
MAD(UFM)	0.92	1	0.95	0.97	1	0.81	0.99	0.96	0.95	0.86	0.94	0.88	0.92
AI(UFXYZ)	0.75	0.81	0.81	0.8	0.81	1	0.8	0.86	0.86	0.79	0.85	0.9	0.9
ENMO	0.96	0.99	0.93	0.96	0.99	0.8	1	0.95	0.94	0.85	0.92	0.86	0.91
HFEN	0.88	0.96	0.95	0.95	0.96	0.86	0.95	1	1	0.87	0.98	0.93	0.97
PIM(FMpre)	0.87	0.95	0.95	0.95	0.95	0.86	0.94	1	1	0.87	0.98	0.94	0.97
ZCM(FMpre)	0.79	0.86	0.91	0.88	0.86	0.79	0.85	0.87	0.87	1	0.9	0.82	0.85
TAT(FMpre)	0.84	0.94	0.96	0.95	0.94	0.85	0.92	0.98	0.98	0.9	1	0.91	0.95
MAD(FMpre)	0.81	0.87	0.86	0.86	0.88	0.9	0.86	0.93	0.94	0.82	0.91	1	0.98
AI(FXYZ)	0.85	0.92	0.91	0.91	0.92	0.9	0.91	0.97	0.97	0.85	0.95	0.98	1

SFig 1. Correlation between the power spectral densities (PSDs) of the activity signals calculated from raw datasets (UFM, UFXYZ) and filtered datasets (FMpre, FXYZ). In addition, we included the PIM metric applied on the UFNM dataset, too. The Pearson's correlation coefficients are calculated by 42 measurements, and their means are represented. Cells in the red square indicate correlations between activity signals calculated by the unfiltered datasets, while cells in the blue square include correlations between activity signals calculated by the filtered datasets. Since our goal is to compare all the 7 metrics, but no filtering is possible in the case of ENMO, and HFEN demands a specially filtered dataset, we included them in both comparisons.
