# Supplementary material for: Detailed analysis and comparison of different activity metrics
Source: PLoS One. 2021 Dec 21;16(12):e0261718. doi: 10.1371/journal.pone.0261718 (PMC8691611; doi:10.1371/journal.pone.0261718)
Supplement: S5 Appendix — (PDF) [file pone.0261718.s006.pdf]

S5 Appendix – Further indicators based on the activity signals determined per axis using TAT and MAD metrics

In the article, Fig 11 presents the analysis of further indicators based on the PIM and ZCM metrics. In addition to this, the following figure presents the same analysis in the case of TAT and MAD metrics.

(a)

	PIM(UFNM)	ENMO	HFEN	PIM(FMpre)	ZCM(FMpre)	TAT(FMpre)	MAD(FMpre)	PIM(|FMpost|)	ZCM(FMpost)	TAT(FMpost)	MAD(FMpost)	AI(FXYZ)
TAT(FMpre)	0.9	0.86	0.97	0.98	0.85	1	0.88	0.91	0.94	0.93	0.91	0.94
TAT(FMpost)	0.96	0.92	0.9	0.91	0.81	0.93	0.75	0.98	0.99	1	0.98	0.84
TAT(FX)	0.89	0.85	0.96	0.97	0.82	0.98	0.88	0.9	0.91	0.91	0.9	0.94
TAT(FY)	0.91	0.88	0.95	0.96	0.8	0.97	0.84	0.93	0.94	0.95	0.93	0.91
TAT(FZ)	0.84	0.81	0.96	0.96	0.81	0.97	0.89	0.85	0.87	0.86	0.85	0.94
TAT(FX)+TAT(FY)+TAT(FZ)	0.9	0.87	0.98	0.99	0.83	1	0.89	0.91	0.93	0.93	0.91	0.95
√(TAT(FX)+TAT(FY)+TAT(FZ))	0.81	0.77	0.93	0.94	0.86	0.95	0.94	0.82	0.85	0.84	0.82	0.96
TAT(FX)^2	0.9	0.88	0.9	0.9	0.68	0.9	0.72	0.9	0.88	0.9	0.9	0.82
TAT(FY)^2	0.91	0.89	0.86	0.87	0.66	0.87	0.67	0.92	0.9	0.92	0.92	0.78
TAT(FZ)^2	0.84	0.81	0.9	0.91	0.67	0.9	0.76	0.84	0.83	0.84	0.84	0.84
TAT(FX)^2+TAT(FY)^2+TAT(FZ)^2	0.93	0.9	0.93	0.94	0.7	0.93	0.75	0.93	0.92	0.93	0.93	0.85
√(TAT(FX)^2+TAT(FY)^2+TAT(FZ)^2)	0.85	0.85	0.79	0.79	0.47	0.76	0.56	0.85	0.78	0.82	0.85	0.69
TAT(FX^2)	0.86	0.86	0.92	0.92	0.6	0.87	0.84	0.86	0.8	0.83	0.86	0.89
TAT(FY^2)	0.9	0.89	0.9	0.9	0.61	0.87	0.79	0.9	0.85	0.88	0.9	0.85
TAT(FZ^2)	0.78	0.77	0.91	0.92	0.61	0.87	0.86	0.78	0.75	0.77	0.78	0.89
TAT(FX^2)+TAT(FY^2)+TAT(FZ^2)	0.9	0.89	0.96	0.97	0.64	0.92	0.88	0.9	0.85	0.88	0.9	0.93
√(TAT(FX^2)+TAT(FY^2)+TAT(FZ^2))	0.84	0.81	0.96	0.97	0.76	0.95	0.97	0.85	0.85	0.85	0.85	0.99

(b)

	PIM(UFNM)	ENMO	HFEN	PIM(FMpre)	ZCM(FMpre)	TAT(FMpre)	MAD(FMpre)	PIM(|FMpost|)	ZCM(FMpost)	TAT(FMpost)	MAD(FMpost)	AI(FXYZ)
MAD(FMpre)	0.77	0.75	0.92	0.92	0.71	0.88	0.77	0.77	0.75	0.75	0.77	0.98
MAD(FMpost)	0.99	0.96	0.92	0.92	0.75	0.91	0.77	1	0.96	0.98	1	0.86
MAD(FX)	0.9	0.88	0.98	0.98	0.77	0.95	0.9	0.9	0.88	0.89	0.91	0.96
MAD(FY)	0.93	0.91	0.97	0.97	0.76	0.95	0.94	0.94	0.92	0.93	0.87	0.93
MAD(FZ)	0.85	0.82	0.97	0.97	0.77	0.95	0.84	0.84	0.84	0.84	0.92	0.95
MAD(FX)+MAD(FY)+MAD(FZ)	0.92	0.89	1	1	0.79	0.98	0.92	0.92	0.9	0.91	0.92	0.97
√(MAD(FX)+MAD(FY)+MAD(FZ))	0.85	0.82	0.97	0.97	0.84	0.96	0.86	0.86	0.87	0.86	0.95	0.98
MAD(FX)^2	0.87	0.88	0.87	0.86	0.54	0.79	0.84	0.84	0.76	0.79	0.73	0.8
MAD(FY)^2	0.89	0.9	0.84	0.84	0.54	0.79	0.88	0.88	0.8	0.83	0.68	0.77
MAD(FZ)^2	0.81	0.81	0.88	0.88	0.57	0.83	0.79	0.79	0.74	0.77	0.77	0.83
MAD(FX)^2+MAD(FY)^2+MAD(FZ)^2	0.91	0.92	0.91	0.91	0.58	0.84	0.89	0.89	0.81	0.84	0.76	0.84
√(MAD(FX)^2+MAD(FY)^2+MAD(FZ)^2)	0.62	0.67	0.56	0.56	0.21	0.45	0.58	0.58	0.44	0.48	0.43	0.5
MAD(FX^2)	0.79	0.81	0.85	0.84	0.51	0.75	0.77	0.77	0.67	0.7	0.84	0.85
MAD(FY^2)	0.84	0.86	0.86	0.86	0.54	0.78	0.83	0.83	0.73	0.77	0.81	0.85
MAD(FZ^2)	0.75	0.75	0.86	0.86	0.55	0.78	0.74	0.74	0.67	0.7	0.86	0.87
MAD(FX^2)+MAD(FY^2)+MAD(FZ^2)	0.84	0.86	0.91	0.91	0.56	0.82	0.83	0.83	0.74	0.77	0.89	0.91
√(MAD(FX^2)+MAD(FY^2)+MAD(FZ^2))	0.82	0.8	0.95	0.95	0.73	0.91	0.82	0.82	0.8	0.81	0.99	1

SFig 1. Extension of the article's Fig 11.
