# Supplementary material for: Detailed analysis and comparison of different activity metrics
Source: PLoS One. 2021 Dec 21;16(12):e0261718. doi: 10.1371/journal.pone.0261718 (PMC8691611; doi:10.1371/journal.pone.0261718)
Supplement: Attachment — Submitted filename: ActigraphyPlosOne_rebuttal_letter.pdf [file pone.0261718.s007.pdf]

Response to peer reviewer comments

First of all, we thank the Reviewers and the Editor very much for reading our manuscript carefully, and for the valuable comments. We have tried to improve our manuscript to be clearer and more suitable for publication in PLOS ONE. In the following we respond to the comments of the Reviewers. Note that we provide a copy of the revised manuscript with track changes to aid following the changes we have made.

Regarding our responses to the comments, we have added new references to the manuscript and we also standardized the style of them. We have utilized the Preflight Analysis and Conversion Engine (PACE) digital diagnostic tool to ensure the figures meet PLOS requirements. In addition, we also corrected the mismatch of the funding information.

Reviewer 1

This paper examines different accelerometer data pre-processing strategies and describes the correlations between a range of resulting metrics (e.g., MAD, ENMO, zero crossings). The paper is interesting in places, but the rationale for performing the work, and the explicit aims could be better articulated. Because these are underdeveloped, it is unclear what the paper sets out to achieve.

Comment 1: *The quality of the English could also be improved, as the paper somewhat jarring in places. Ideally it would have been proofread by someone proficient in written English prior to submission (to make it easier for the reviewers to focus on the science).*

Response: We agree that the language of our paper could be improved. The text has been modified with the intention to meet the reviewer's standards.

Action: According to the reviewer's comment we improved the manuscript in several places.

Comment 2: *The rationale in the introduction is not well articulated. I feel several parts of the introduction do not contribute towards the rationale and can be removed. E.g., L56-69 describes the failure to report the key aspects of study methodology, and L46-55 is about sleep disorders. The link between these ideas and different accelerometer pre-processing decisions is not clear.*

Response: We concur with the reviewer that the presentation of the frequent and successful uses of actigraphy is achievable with a much more factual listing in the L46-55 sentences. In the new version, we are summarizing the areas of use without putting that much emphasis on sleep disorders. We are also in agreement with the reviewer that the logical connection between L46-55 and L56-69 can be better articulated. In the new version, we more precisely illustrate the duality: as presented in the L46-55 paragraph, although actigraphy is a commonly used and versatile method, it is utilized without a uniform methodology and generalization which is the cause of all those uncertainties about the activity determination procedure presented in L56-69 (for example, the possible preprocessing decisions, which will be explained in more details in our response of the next comment), and these issues are independent of the scientific fields of use, therefore we construe them in an extensive and general way.

Action: The presentation of the different fields of use of actigraphy ***(L46-54 of the updated manuscript)*** has been shortened by taking less emphasis on sleep disorders.

"One of the most common fields of use of actigraphic recordings is sleep medicine since, by analysing the activity signal, scientists can reliably characterize the subjects' sleep quality [10] and possible disturbance in their circadian rhythm [11], which are commonly related to mental disorders [12]. Therefore, actigraphy is also frequently utilized in psychiatric research; for example, to recognize behavioural disorders or to distinguish between similar mental diseases [13]. Another immensely current use of actigraphs is to determine human physical activity (PA) [4]. In addition to their therapeutic usage, actigraphic devices are also successfully utilized in the field of human motion pattern analysis [5,6]; for example, to find regularities in the distribution of humans' resting and active period durations."

We also rewrote the next paragraph ***(L55-60 of the updated manuscript)*** to more precisely illustrate the logical connection between the presentation of the fields of uses ***(L46-54 of the updated manuscript)*** and the following parts ***(L61-96 of the updated manuscript)*** of the "Introduction" chapter.

"Since actigraphy is a widely and regularly applied multidisciplinary method, its high degree of standardization and generalization is required and expected. Conversely, multiple studies have already pointed out that the utilization of actigraphs is inconsistent, independently of the field of use. As a consequence, direct comparisons of findings between different studies and the exact reproduction of the results are problematic in several cases. In the following, we explain this in an extensive and general way."

Comment 3: *Given the main aim of this study is to compare different metrics obtained from different pre-processing decisions, the authors fail to acknowledge that the vast majority of researchers do not perform any pre-processing themselves - they simply use the output provided by the manufacturer's software, which in most cases is well documented (e.g., Actigraph, Actical, ActivPAL, Axivity, GENEActiv).*

Response: We agree with the reviewer that this phenomenon is caused by the fact that researchers understandably use commercially available devices to generate activity signals, so in their articles, they only specify what tool they used. We also agree that this must be better emphasized in the "Introduction" chapter. However, we would argue that the discussed preprocessing steps would be well documented in the case of all the mentioned devices. The problem is exactly what we are trying to explain better in the new version: many steps and parameters are not known for multiple devices. For the most common actigraphic device manufacturer (ActiGraph) related to physical activity researches, that is also mentioned by the reviewer: they use a bandpass filter to preprocess the raw acceleration signal, yet based on the manufacturer documentation the filter's order is unknown (an independent study tried to answer this with reverse engineering [1]). The same is true for the exact way how these devices calculate their proprietary Activity Count (AC) metric.

Several other manufacturer's devices that are listed as examples by the reviewer measure just the raw acceleration signals, so it is up to the user to perform the preprocessing and the determination of the activity values, as well as to document these steps with sufficient precision in their studies. The general lack of sufficient documenting is mentioned in our L56-69 sentences (for example, the lack of stating the applied threshold values for the Zero-Crossing Method and Time Above Threshold activity metrics). Even if manufacturers provide utilities (for example, software packages) to process the raw acceleration data, various preprocessing and activity calculation steps are available in these utilities that differ by manufacturers (for example, the filtering of the raw acceleration signal is not supported in the GENEActiv software package [2], while the Axivity manufacturer recommends several filtering methods in their supporting documentation [3]). These conflicts enhance the diversification of activity calculation procedures across studies that is contradictory to standardization and makes both the reproducibility of

the results and comparability of different studies' results difficult, especially if the given procedure is not precisely documented either by the manufacturer or the researcher. Furthermore, looking ahead to one of the reviewer's criticisms about the lack of some aspects of the specification on our custom-made actigraphic device: this critic is more pertinent in the case of commercially available and frequently used actigraphic devices.

Before our article, several studies and review articles have criticized this and encourages us to generalize the use of actigraphy. Let us sum up their corresponding statements. In a physical activity-related study [4], that is published in PLOS ONE, the authors engross their concerns about the fact that the activity signals do not truly represent the raw acceleration signals from which they were derived, and since the activity value determination procedures are closely guarded proprietary algorithms that vary by manufacturer, the activity data obtained from different actigraph brands and models are generally not comparable. In the Movisense actigraph manufacturer's whitepapers [5], it is stated that the raw acceleration data can be processed in a wide range, which frequently includes signal filtering techniques, however, the filter properties depend on the manufacturer and vary which makes it nearly impossible to compare the activity values generated by different companies' devices yet they are commonly and similarly called as "counts". Another study [6] related to sleep medicine also affirms that activity data is commonly reported as "activity counts" even though they are determined by different devices and produced in a very difficult way. In a physical activity-related review article [7], the authors highlight that actigraphic measurements are not nearly well documented, for example almost 80% of the studies they examined do not report any information on the used filter. Multiple studies [8,9] further criticize the reporting complexity of studies, hence they frequently inconsistently report key aspects of the utilization of actigraphic devices, such as device type, sampling rate, epoch length, data processing, software. Their conclusions are all identical: actigraphic devices determine activity values in such a diverse and insufficiently documented way, that reproduction of results and comparison between studies are nearly impossible. And beyond this, if someone uses such a device that records the raw acceleration signal, even if it is a commercially available device, the obvious question arises; what tools are in our repertoire and how to use them to determine the activity values and what are the differences between the different possible procedures?

Action: We completely rewrote the problem statement part ***(L61-96 of the updated manuscript)*** of the "Introduction". We extended the description of the inconsistency in the actigraphic literature similarly to our response. We made an addition based on the reviewer's remark about how scientists use these devices. The complexity of our problem statement has been increased by referring to two new manufacturers (GENEActiv, Axivity) that are part of the reviewer's examples. This section has been split into multiple paragraphs for better perspicuity.

Comment 4: *What would really strengthen the rationale is a thorough description of how understanding the correlations between metrics derived from raw could be useful to researchers? The aim(s) could be presented much more explicitly, as there are several parts to the results (e.g., identifying thresholds, correlations, further divided into pre-processing, different activity metrics, axes).*

Response: We agree with the reviewer that in the article we did not emphasize as much as we could how this article can be useful for the researchers, and our aims could be presented more clearly. We have now modified the article, accordingly. In the new version of the "Introduction" chapter, we also emphasized that this study is not just another review article which highlights the issues of the lack of standardization,

but also informs the readers about the possibilities to calculate activity values, and helps them to select an appropriate activity determination procedure. To facilitate this choice, we assessed how the collected preprocessing methods and activity metrics can be combined, while we also examined their effect on the resulting activity signals.

Action: We modified the corresponding part of the "Introduction" ***(L97-102 of the updated manuscript)*** to better articulate why this work could be useful for researchers.

"The aim of this study is not only to highlight these issues again, but to examine the effect of the processing steps leading from the raw acceleration signals to the activity signals with the purpose to help scientists to explore, differentiate and choose between the diverse activity calculation possibilities, which is especially helpful if their device records the raw acceleration signal. In the following, we are summarizing those questions that generally arise during the activity value determination process."

Comment 5: *P3L37: What do you mean by 'actigraph'. Is this the device developed by the Actigraph company? Or broadly speaking a wearable device that contains an accelerometer?*

Response: We thank the reviewer for pointing out that our terminology may sound complicated for the reader. On the one hand, we used the term "actigraph" independently of the scientific field of use, the manufacturer, or the exact device type; any wearable device that logs the acceleration or records the locomotor activity signal of the body part that is placed on is named as actigraph. On the other hand, when we meant to talk about the similarly named company, we used the same formatting as they do officially: "ActiGraph". That is because we found this naming scheme is conventional in the literature [10,11]. However, we understand that it may sound blurred for the readers, therefore we clarified it in the new version at the beginning of the "Introduction" chapter to prevent any misunderstandings.

Action: We have modified the introduction of actigraphic devices ***(L37-40 of the updated manuscript)*** to clarify what devices we call as "actigraphs" in general.

"The method applies a small, acceleration sensor-based, non-invasive diagnostic tool which is called actigraph, independently of the manufacturer. The actigraph, worn on the non-dominant wrist or sometimes on the hip, can objectively, reliably and cost-effectively record the locomotor activity of the subject."

Comment 6: *P3L46: I wouldn't say actigraphy is a method in polysomnogrphy. The validity of using accelerometer data for true measures of sleep quality is also questionable.*

Response: The reviewer made an important comment about our superficial phrasing, which we agree with. Due to the reviewer's second comment, we already modified this section and removed this part.

Action: We already modified that part of our text where this statement was ***(L46-49 of the updated manuscript)***, and we removed it corresponding to the action on the reviewer's second comment.

Comment 7: *P3L54: What do you mean by 'important results'. This is very ambiguous.*

Response: We agree with the reviewer about this concern. In the updated version, we have clarified it by giving an example.

Action: We removed the phrase "important result" from that sentence in question ***(L51-54 of the updated manuscript)***, and we gave an example instead.

“In addition to their therapeutic usage, actigraphic devices are also successfully utilized in the field of human motion pattern analysis [5,6]; for example, to find regularities in the distribution of humans’ resting and active period durations.”

Comment 8: *The headings in this section are somewhat misleading. For example, the 'Measurement data' heading is about participants and recruitment, not about the data.*

…

L144¬, L170, L227 etc: These sections read more like a literature review than a succinct description of the methods, making it difficult to differentiate between theory and what was performed in this study.

Response: We thank the reviewer for highlighting these, let us answer all the questions regarding the chapter naming and organization at once. When creating our headings, we followed PLOS ONE’s manuscript organization rules [12] which allows the writer to rename or reorganize the middle section’s elements freely. We understand that the “Measurement data” naming not exactly reflect the content of this section, therefore we renamed it.

The reviewer is right that the L144, L170, L227 sections read like a literature review, as it was necessary to introduce all the different preprocessing possibilities and activity metrics that are then the objects of our analysis. In the updated version we highlighted that these methods are presented with a purpose since these are then comprehensively compared during our analysis. However, we would like to highlight to the reviewer that this chapter which we call “Methods of determining activity values” has a different purpose than the, generally speaking, “Methods” chapter of other studies. Our equivalent to that kind of “Methods” section is titled “Examination methods”. To resolve this, we renamed these chapter titles in the updated version.

Action: We renamed the “Measurement data” to “Actigraphic recordings”, “Methods of determining activity values” to “Common procedures of determining activity values”, and “Examination methods” to “Methods of examination”.

We concretized why this review part was necessary in our work ***(L213-215 of the updated manuscript)***.

“*Before we introduce our examination methods, we are summarizing the different preprocessing steps and activity metrics which could be found in the literature so one can see what issues arise during preprocessing and activity calculation if we have digitized acceleration signals.”*

We also clarified that the naming scheme of the datasets were introduced by us ***(L221-222 of the updated manuscript)*** and is not taken over from the literature.

“The possible dataset types that result from the preprocessing step are reviewed below with a nomenclature introduced by us.”

Comment 9: *P6L112-118: It sounds like this was a custom-made device. You should provide information on the validity and reliability of the acceleration output and RTC. Is there any drift in the timestamp, and is the sampling frequency constant or does it fluctuate?*

Response: We agree that there was missing important information about the reliability and accuracy of the developed device. We have examined the specifications of several commercially available devices (Fitbit, Actical, Axtivity AX3, GENEActiv Original and „GENEA”, ActiGraph GT3X/GT3X+/wGT3X-BT/GT1M).

Surprisingly, the timing accuracy is given only for two devices (no sampling frequency fluctuation is specified for any device), and it was not easy to find out what kind of sensor they use (in order to get more complete performance information). Our device uses a high-performance MEMS acceleration sensor with very similar specifications. This sensor is intended to be used in motion sensing applications and provides both the user-programmable sampling rate and the accurate analogue-to-digital conversion. Since the microcontroller receives digital data from the sensor, the overall accuracy of the device is the same as the accuracy of the sensor and the fully detailed specifications are given in the freely available datasheet. The additional integrated real-time quartz clock ensures long term timing accuracy with a tolerance of +/- 20 ppm. Therefore, by making the specifications more detailed in the paper and referencing the sensor's full specification means that our device is probably the most completely specified one and it provides proper accuracy and reliability – we thank the reviewer to draw our attention to this important point.

Action: We have modified and extended the corresponding section ***(L143-158 of the updated manuscript)*** to allow the reader to have the most complete information about the accuracy, reliability and validity of the used device.

"We have developed a small device housed in a 3D-printed capsule of size 41 mm × 16 mm × 11.3 mm (LWH) which weighs only 5.94 grams. The device fits in a commercially available wristband, integrates a microcontroller (C8051F410), a 3-axis MEMS acceleration sensor (LIS3DH) and a 1GB-capacity flash memory chip. The high-performance acceleration sensor is intended to be used in motion sensing applications and provides both the user-programmable sampling rate and the accurate analogue-to-digital conversion. Since the microcontroller receives digital data from the sensor, the overall accuracy of the device is the same as the accuracy of the sensor. The detailed specifications are given in the freely available datasheet. The device incorporates a real-time quartz clock as well to ensure long-term timing accuracy with a tolerance of +/- 20 ppm.

The microcontroller continuously reads the data from the accelerometer chip, whose sampling rate can be set from 1 sample/sec to 100 samples/sec. At the used rate of 10 samples/sec, the device is capable of recording raw acceleration data continuously into the device's flash memory for more than three weeks. According to the above, our solution provides all the features required for the acquisition of the acceleration signals, just as the commercially available actigraphs. In addition, we have full control over the measurement process, all the specifications are openly accessible, and any kind of signal processing can be implemented."

Comment 10: *P6: A fundamental point here is that given you have used a device that has not been used in any other physical activity research, how can you expect your results to be generalisable other literature?*

Response: As we have explained above, our device captures the raw acceleration signals using a dedicated high-performance sensor that provides as accurate data as the commercially available actigraph devices. The quality of the recorded acceleration signals is guaranteed by the digital output acceleration sensor, it meets the requirements (for details, please see our response to the previous comment). Since our device does not perform any preprocessing on the acceleration signal, any kind of activity metrics based on the acceleration signals can be computed regardless of the aim of the research. One of the main advantages of our solution is that our device provides the most complete information about the activity process, the device does not perform any data reduction with any kind of pre-processing. Accordingly, any activity calculation method can be reproduced on the recorded signal with the same accuracy and reliability as if

using a commercially available device. Our paper focuses on the problem of the incompletely specified preprocessing of the commonly used actigraphs in many aspects, so we address this issue in several places both in the article and in our responses.

Please see our answer above where we have pointed out that our device is compatible with the commercially available actigraph devices.

Action: Please see the action description at the previous comment. Several other modifications in the paper are related to this point due to the aim of comparing the activity calculation methods.

Comment 11: *P6L123: Why were participants with impaired neurocognitive function excluded? This seems strange given the main aim is to compare accelerometer metrics (where greater heterogeneity in your dataset is probably useful).*

Response: We agree with the reviewer that a heterogeneous group of participants is advantageous in our case. However, such extreme cases (for example bipolar disorder, epilepsy) that could affect our results must be excluded. Our group of participants was recruited for a large-scale psychiatry-related study, where it was crucial to study only healthy subjects. That is the reason behind this kind of participant recruitment process. We would note that only two individuals were excluded due to existing mental disorders, but none due to neurocognitive dysfunction during the selection process. The existence of such a healthy group of subjects came in line with our needs, as irregular mental conditions could affect the physical behavior of the subject, which in turn would have affected our results, too. Nonetheless, it would be interesting to examine whether these various mental conditions can be detected from the activity signals, but this is an existing research field for actigraphy, and that was not our goal. We aimed to create a comparative analysis of the different activity calculation procedures in a technical aspect where the acceleration signals were measured on a heterogeneous group of healthy human subjects to reduce the likelihood of mental conditions interfering with the outcome of our investigations. In addition, if we would have measured non-healthy subjects, too, it would have required a much complex selecting strategy for their recruitment and we would have obtained a much more difficult to reproduce a group of subjects.

Action: We extended the corresponding part of that section ***(L165-168 of the updated manuscript)*** with the following.

"With this selection strategy, such a heterogenous group of healthy participants was generated that is easily reproducible, and reduces the possibility that extreme cases (for example, patients with mental disorders or epilepsy) impact our results."

Comment 12: *P6L127: Why was 10 Hz and 8 g chosen? This is a fundamental decision that needs to be justified in relation to the purpose of the study. Why didn't you use 30 Hz ±6 g or 100 Hz ±8 g which are much more common in physical activity literature. A different sample frequency and measurement range will change the results, which has implications for generalisability.*

Response: We agree with the reviewer that our decision regarding the selection of sampling rate and measurement range should be justified by us. Before answering, we would like to highlight our objective which is more precisely stated in the article's updated version: actigraphy measurements are not limited to physical activity research, and we followed a more general approach that is not restricted to a specific field. Actigraphs in sleep and circadian rhythm-related studies are frequently utilized with 10 Hz sampling rate [13–15] while bipolar disorder episodes can be classified by analyzing the activity values that are

calculated by acceleration data that is recorded with 6 Hz sampling rate [16]. We concur that in the field of physical activity research higher than 10 Hz sampling rates are more commonly selected, however, PA studies have shown that the classification of sedentary activities, household activities, walking, and running can be achieved using the raw acceleration data sampled at 10 Hz using only one axis [17] and there is no significant statistical difference between ActiGraph's activity counts between 10 Hz and 30 Hz sampling rate [18]. Nonetheless, measuring at a higher rate has a high impact on the maximum measurement length. We concluded that the 10 Hz and ±8 g measurement range is appropriate for general-purpose free-living activity recordings considering the fact that 10 Hz measurements are acceptable on diverse fields and longer duration measurements can be achieved in this way. Regarding the measurement range, the maximum range is limited by the acceleration sensor in general. In the case of the commercially available ActiGraph devices, older models support only ±3 g and ±5 g measurement intervals, while newer models are capable of recording acceleration in the range of ±8 g simply because these are the limits of those sensors. Similarly to the current flagship model from ActiGraph (wGT3X-BT), our sensor can record ±8 g acceleration. Therefore, we used this in our configuration.

Action: We extended the corresponding paragraph ***(L169-171 of the updated manuscript)*** of the newly titled "Actigraphic recordings" section with a description why we decided to use 10 Hz sampling rate.

"The collected free-living acceleration signals were sampled at 10 Hz in the ±8 g measurement interval, as this configuration is viable in diverse fields of use [1,28–32], and our study is not restricted to any specific discipline."

Comment 13: *P15L329: More information is needed to describe the main analysis (Pearson's correlation). It is currently unclear what the unit of observation is (i.e., is it the 42 subjects, or is it the epoch data, meaning you have repeated measures for each subject). The number of observations in this analysis is not shown in the results.*

Response: We are thankful to the reviewer for raising our awareness about this unclearness. Each 10-days-long raw triaxial data is measured on a different subject. We calculated 148 different activity signals based on the combination of different preprocessing methods and activity metrics from a single subject's recording. We then calculated the Pearson's correlation coefficient between these activity signals that resulted in a correlation matrix. We did this in the case of every subject, to get a total of 42 correlation matrices. Then we calculated the mean and standard deviation of these matrices' identically located cells. As a result, we reduced the 42 matrices to a single, averaged correlation matrix. By analyzing this final matrix, we could assess how the differently produced activity signals correlate to each other based on 42 different subjects' 10-days-long motions. In the modified version of this article, we clarified the description of the main analysis.

Action: We rewrote the description ***(L375-382 of the updated manuscript)*** of our correlation analysis at the newly titled "Methods of examination" section. The new text is basically a step-by-step description about what kind of correlations we calculated, and how did we create the single correlation matrix that is also part of the supporting information.

"We determined all the 148 possible activity signals from a single subject's 10-days-long recording. Then we computed the Pearson's correlation coefficients between the activity signals, which resulted in a correlation matrix. It was performed on all the 42 subjects to obtain 42 correlation matrices. Then we reduced these matrices to a single, averaged matrix by calculating the mean and standard deviation of the

identical cells of the 42 matrices. By analysing the averaged correlation matrix, we could observe how the different activity calculation procedures correlate with each other, based on 42 different subjects' 10-days-long acceleration recordings."

Reviewer 2

This is an important paper which should be intensively studied by all the scientists who begin with actigraphic measurements and analysis. It serves both as a critical survey of the methods used in the field and with proposed solutions to cure the deficiencies of the available methods.

Comment 1: *I propose the publication of the paper after a minor addition about the claim that the gravitational correction can also be done by filtering. I don't really get that because, it seems, in spacial cases, the gravitational component can be in he same frequency range as the activity signal. Example: the human object is rotating his/her wrist in a periodic fashion.*

Response: We thank the reviewer for this comment. This filtering technique is not introduced by us, we have taken it over from the literature. However, we agree that in special cases the hand movement's frequency can be in the same frequency range as the gravitational component, and this kind of bandpass filtering can also eliminate specific low-frequency movements of the subject next to the gravitational component. Because of this concern, we also examined another preprocessing method. The gravitational component can be eliminated without filtering if we subtract 1 g from the magnitude of acceleration and then we take the absolute value of the difference. This preprocessing is so-called normalization. However, the two preprocessing have different usages: the normalization can be applied only on the magnitudes of acceleration, while the filtering can be done per axis, too, so both of them are included in our comparative analysis. In the updated version, we extended the description of the normalization that it is useful to eliminate the effect of gravity of Earth without the risk of losing low-frequency components of the hand movement.

Action: We have extended the description of the normalization method ***(L250-252 of the updated manuscript)*** according to our response.

"In contrast to the filtering-based preprocessing, with normalization, we can eliminate the g without the risk of losing those low frequency components of the hand movement that are in the same frequency range as the gravity of Earth (i.e., below 0.25 Hz)."

References in our responses

[1] Brønd JC, Andersen LB, Arvidsson D. Generating ActiGraph Counts from Raw Acceleration Recorded by an Alternative Monitor. Med Sci Sports Exerc 2017;49:2351–60. https://doi.org/10.1249/mss.0000000000001344.

[2] GENEActiv - GENEAread R Package 2020. https://github.com/cran/GENEAread (accessed October 27, 2021).

[3] Ploetz T, Hammerla N. Axivity - Standard Procedures for (Wrist Worn) Accelerometry 2014. https://axivity.com/files/resources/StandardProceduresforAccelerometry.pdf (accessed July 27, 2021).

[4] Duncan S, Stewart T, Bo Schneller M, Godbole S, Cain K, Kerr J. Convergent validity of ActiGraph and Actical accelerometers for estimating physical activity in adults. PLoS ONE 2018;13:e0198587. https://doi.org/10.1371/journal.pone.0198587.

[5] Richert F. movisens GmbH - Activity Monitoring and Existing Algorithms Regarding Energy Expenditure Estimation: A Short Review 2017. https://www.movisens.com/wp-content/downloads/WPMove3_Activity_Algorithms.pdf (accessed June 24, 2021).

[6] Ancoli-Israel S, Cole R, Alessi C, Chambers M, Moorcroft W, Pollak CP. The role of actigraphy in the study of sleep and circadian rhythms. Sleep 2003;26:342–92. https://doi.org/10.1093/sleep/26.3.342.

[7] Migueles JH, Cadenas-Sanchez C, Ekelund U, Delisle Nyström C, Mora-Gonzalez J, Löf M, et al. Accelerometer Data Collection and Processing Criteria to Assess Physical Activity and Other Outcomes: A Systematic Review and Practical Considerations. Sports Med 2017;47:1821–45. https://doi.org/10.1007/s40279-017-0716-0.

[8] Berger AM, Wielgus KK, Young-McCaughan S, Fischer P, Farr L, Lee KA. Methodological Challenges When Using Actigraphy in Research. Journal of Pain and Symptom Management 2008;36:191–9. https://doi.org/10.1016/j.jpainsymman.2007.10.008.

[9] Meltzer LJ, Montgomery-Downs HE, Insana SP, Walsh CM. Use of actigraphy for assessment in pediatric sleep research. Sleep Med Rev 2012;16:463–75. https://doi.org/10.1016/j.smrv.2011.10.002.

[10] Smith C, Galland B, Taylor R, Meredith-Jones K. ActiGraph GT3X+ and Actical Wrist and Hip Worn Accelerometers for Sleep and Wake Indices in Young Children Using an Automated Algorithm: Validation With Polysomnography. Frontiers in Psychiatry 2020;10:958. https://doi.org/10.3389/fpsyt.2019.00958.

[11] Martin JL, Hakim AD. Wrist actigraphy. Chest 2011;139:1514–27. https://doi.org/10.1378/chest.10-1872.

[12] PLOS ONE Submission Guidelines n.d. https://journals.plos.org/plosone/s/submission-guidelines (accessed October 27, 2021).

[13] Fekedulegn D, Andrew ME, Shi M, Violanti JM, Knox S, Innes KE. Actigraphy-Based Assessment of Sleep Parameters. Ann Work Expo Health 2020;64:350–67. https://doi.org/10.1093/annweh/wxaa007.

[14] Natale V, Plazzi G, Martoni M. Actigraphy in the Assessment of Insomnia: A Quantitative Approach. Sleep 2009;32:767–71.

[15] Tsanas A, Woodward E, Ehlers A. Objective Characterization of Activity, Sleep, and Circadian Rhythm Patterns Using a Wrist-Worn Actigraphy Sensor: Insights Into Posttraumatic Stress Disorder. JMIR Mhealth Uhealth 2020;8:e14306. https://doi.org/10.2196/14306.

[16] Cuesta-Frau D, Schneider J, Bakštein E, Vostatek P, Spaniel F, Novák D. Classification of Actigraphy Records from Bipolar Disorder Patients Using Slope Entropy: A Feasibility Study. Entropy 2020;22:1243. https://doi.org/10.3390/e22111243.

[17] Zhang S, Murray P, Zillmer R, Eston RG, Catt M, Rowlands AV. Activity classification using the GENEA: optimum sampling frequency and number of axes. Med Sci Sports Exerc 2012;44:2228–34. https://doi.org/10.1249/mss.0b013e31825e19fd.
[18] John D, Tyo B, Bassett DR. Comparison of four ActiGraph accelerometers during walking and running. Med Sci Sports Exerc 2010;42:368–74. https://doi.org/10.1249/mss.0b013e3181b3af49.
