# Supplementary material for: Detailed analysis and comparison of different activity metrics
Source: PLoS One. 2021 Dec 21;16(12):e0261718. doi: 10.1371/journal.pone.0261718 (PMC8691611; doi:10.1371/journal.pone.0261718)
Supplement: Attachment — Submitted filename: ActigraphyPlosOne_rebuttal_letter2.pdf [file pone.0261718.s008.pdf]

Response to peer reviewer comments

First of all, we are pleased to see that both Reviewers expressed that the manuscript has improved sufficiently, and we have answered properly all but one of their raised questions. In our response, we are replying to the only remaining remark of the first Reviewer.

Reviewer 1

The authors have made extensive revisions to the original submission, and the paper has definitely improved, so well done. In general, I still think the text is overly verbose throughout and could be structured much more succinctly.

Comment 1: *One point I will make is I still do not understand the correlation analysis method. It sounds like you obtained a correlation matrix separately for each subject. Specifically, a 148x148 correlation matrix, for each of the 42 subjects (which were later averaged). I don't know how many observations were used in each correlation. As you had 10 days, did you summarise each of the 148 activity signals per day, so each individual correlation had 10 paired data points? Or were these correlations performed using the raw 10 Hz data (~8.6m data points across 10 days)?*

Response: From a single subject's 10-days-long raw acceleration signal, we determined an activity value for each 1-minute-long epoch, so that a 10-days-long acceleration signal (approx. 8.6 million datapoints) became a minutely-sampled, 10-days-long activity signal (approx. 14000 datapoints). We executed this minute-based activity calculation process in 148 different ways based on the different preprocessings and activity metrics. We compared the resulting 148, minutely-sampled activity signals in a 148×148 correlation matrix for each subject. Then we reduced the 42 correlation matrices into a single correlation matrix by averaging their identically located cells. A cell of the averaged matrix indicates the average correlation of two differently calculated activity signals based on 42 different subjects' 10-days-long recordings.

Action: Previously, we have already modified the corresponding part of the original manuscript to clarify our method, but we further extended it as seen below **(L365-376 of the updated manuscript)**.

"It can be seen that the obtained activity signal depends on the preprocessing of the acceleration data, as well as on the activity metrics used and on how it is applied to the axial measurements. Our aim is to examine in detail how and to what extent they depend on these steps. Based on the different preprocessings and activity metrics used, we determined all possible (a total of 148) minutely-sampled activity signals from a single subject's 10-days-long raw acceleration data. Then we computed the Pearson's correlation coefficients between these minutely-sampled activity signals to assess their similarity [8,50], which resulted in a 148×148 correlation matrix. It was performed on all the 42 subjects to obtain 42 correlation matrices. Then we reduced these matrices to a single, averaged matrix by calculating the mean and standard deviation of the identically located cells of the 42 matrices. By analysing the averaged correlation matrix, we could observe how the different activity calculation procedures averagely correlate with each other, based on 42 different subjects' 10-day-long recordings."

Comment 2: *The reason why I asked about this in the last review is because a Pearson's correlation is not an appropriate method of statistical analysis if your observations are related. This should only be used when your observations (i.e., rows in your dataset) are independenlt. There are other statistical methods that are appropriate for determining association when the data points are related.*

Response: In our investigation, we did not use Pearson's correlation coefficient for statistical analysis. It is only used to characterize the similarity of the indicators; therefore, statistical independence is not required in this case. Pearson's correlation is an accepted measure of similarity between two time-dependent signals [1–5].

We would like to highlight a few highly relevant studies as examples where the correlation coefficients are used to measure the similarities of time series, as we did.

- Two studies [6,7] published in PLOS ONE and Scientific Reports measured the degree of similarity of time-dependent 1-minute-sampled activity signals by calculating the Pearson correlation coefficients between them identically to our case.
- Another study [8] also published in PLOS ONE utilized the Pearson correlation coefficients to assess the similarity of the time-dependent evolutionary dynamics of different air pollutants.
- Without detailing, further studies [9–12] also use correlation as the measure of similarity between time series.

Action: We have extended the corresponding text by referencing directly related works where the Pearson's correlation coefficients were used as a measure of time series similarity. The updated text can be seen in the action for the previous comment.

References in our responses

[1] Lhermitte S, Verbesselt J, Verstraeten WW, Coppin P. A comparison of time series similarity measures for classification and change detection of ecosystem dynamics. Remote Sensing of Environment 2011;115:3129–52. https://doi.org/10.1016/j.rse.2011.06.020.

[2] Warren Liao T. Clustering of time series data—a survey. Pattern Recognition 2005;38:1857–74. https://doi.org/10.1016/j.patcog.2005.01.025.

[3] Durante F, Pappadà R, Torelli N. Clustering of financial time series in risky scenarios. Adv Data Anal Classif 2014;8:359–76. https://doi.org/10.1007/s11634-013-0160-4.

[4] Derrick T, Thomas J. Time Series Analysis: The Cross-Correlation Function. Innovative Analyses of Human Movement 2004;Chapter 7:189–205.

[5] MathWorks - Measuring Signal Similarities, MATLAB & Simulink Example n.d. https://www.mathworks.com/help/signal/ug/measuring-signal-similarities.html (accessed December 1, 2021).

[6] Bammann K, Thomson NK, Albrecht BM, Buchan DS, Easton C. Generation and validation of ActiGraph GT3X+ accelerometer cut-points for assessing physical activity intensity in older adults. The OUTDOOR ACTIVE validation study. PLOS ONE 2021;16:e0252615. https://doi.org/10.1371/journal.pone.0252615.

[7] Smith MP, Horsch A, Standl M, Heinrich J, Schulz H. Uni- and triaxial accelerometric signals agree during daily routine, but show differences between sports. Sci Rep 2018;8:15055. https://doi.org/10.1038/s41598-018-33288-z.

[8] Dai Y-H, Zhou W-X. Temporal and spatial correlation patterns of air pollutants in Chinese cities. PLOS ONE 2017;12:e0182724. https://doi.org/10.1371/journal.pone.0182724.

[9] Tenenbaum JN, Havlin S, Stanley HE. Earthquake networks based on similar activity patterns. Phys Rev E 2012;86:046107. https://doi.org/10.1103/PhysRevE.86.046107.

[10] Tirabassi G, Sevilla-Escoboza R, Buldú JM, Masoller C. Inferring the connectivity of coupled oscillators from time-series statistical similarity analysis. Sci Rep 2015;5:10829. https://doi.org/10.1038/srep10829.

[11] Rehfeld K, Kurths J. Similarity estimators for irregular and age-uncertain time series. Clim Past 2014;10:107–22. https://doi.org/10.5194/cp-10-107-2014.

[12] Bonanno G, Caldarelli G, Lillo F, Miccichè S, Vandewalle N, Mantegna RN. Networks of equities in financial markets. The European Physical Journal B - Condensed Matter 2004;38:363–71. https://doi.org/10.1140/epjb/e2004-00129-6.
